# Multimodal Psychotherapeutic Inpatient Therapy of Depression Is Successful in Patients With High Cytokine Production

**DOI:** 10.3389/fpsyt.2020.571636

**Published:** 2020-11-02

**Authors:** Eva M. J. Peters, Melanie Neusetzer, Secil Akinci, Aysenur Murat, Sabine Treuherz, Matthias Rose, Frank Leweke, Falk Leichsenring, Melanie L. Conrad, Johannes Kruse

**Affiliations:** ^1^Psychoneuroimmunology Laboratory, Department of Psychosomatics and Psychotherapy, Justus-Liebig-University of Gießen, Gießen, Germany; ^2^Division for General Internal Medicine, Psychosomatics and Psychotherapy, Charité Center 12 Internal Medicine and Dermatology, Charité Universitätsmedizin Berlin, Berlin, Germany; ^3^Department of Psychosomatics and Psychotherapy, Justus-Liebig-University of Gießen, Gießen, Germany; ^4^Charité Center 5 Laboratory and Preventive Medicine, Institute of Microbiology, Infectious Diseases and Immunology, Universitätsmedizin Berlin, Berlin, Germany

**Keywords:** multimodal psychotherapeutic inpatient therapy, stress, depression, tumor necrosis factor alpha (TNFα), inflammation

## Abstract

**Objective:** In experimental settings, systemically elevated inflammation markers interfere with major depression treatment. In German healthcare, compulsory national health insurance covers treatment of a wide variety of depressive disorders, if it follows evidence-based medicine guidelines combining recommended therapies. To date, little is known about the relevance of immune system cytokine production with regard to real-world clinical care for patients with moderate depression.

**Methods:** Seventy three patients with moderate depression subjected to multimodal psychotherapeutic inpatient therapy (mPT) following a psychodynamic concept at a German university hospital were included. As a primary outcome, mPT success, evidenced by delta HADS “depression,” was analyzed according to tumor necrosis factor alpha (TNFα) production by peripheral blood mononuclear cells (PBMC) after phytohemagglutinin (PHA) challenge at baseline. Secondary outcomes addressed the inflammatory response and mental health comparing high and low TNFα-producers.

**Results:** First, higher PBMC TNFα production at baseline predicted a better mPT-outcome (*R*^2^ 0.162, *p* = 0.014). Second, patients with high TNFα (hTNF) at baseline produced significantly more acute inflammatory cytokines [interleukin (IL)1β, IL6), TH1/TH2 cytokines [interferon gamma (IFNγ), IL4] as well as eotaxin and IL2 compared to low TNFα producers (lTNF) (Cohen's ds between −0.532 and −1.013). Demographic data, diagnosis subtype-distribution, medication, systemic inflammation markers [C-reactive protein (CRP), high mobility group box 1 (HMGB1), leptin], anxiety and depression (HADS) did not differ. From baseline to mPT-discharge, HADS “depression” decreased in both hTNF (11.31 to 5.47, p = 0.001, d = 1.184) and lTNF patients (11.50–7.92, *p* = 0.001, *d* = −0.765), while PBMC cytokine production decreased significantly in hTNF (Cohen's ds between −0.304 and −0.345) with a significant group by time interaction for TH1/TH2 ratio. At the end of therapy, comparison of TNF groups revealed significantly lower depression-scores in hTNF compared to lTNF patients (5.47 compared to 7.92, *p* = 0.035, d = 0.504).

**Conclusions:** Our study demonstrates successful treatment of depression in a clinical care setting using multimodal psychotherapy based on a psychodynamic concept following guideline recommendation. The greatest improvement in patient depression was linked to the highest production of TNFα by PBMCs at baseline. Our study contributes to the definition of patient subpopulations with differing cytokine responses that are related to succesful treatment of depression.

## Introduction

The interaction between depression and inflammation is an intensely debated topic (1–8). Evidence is accumulating that stress-exaggerated inflammation can contribute to the development of depression and at the same time promote infections and non-communicable diseases such as auto-immune, metabolic or cardio-vascular disease (9–14). Such maladaptive inflammatory activity is therefore held partly responsible for the frequent co-morbidities that accompany depressive disorders and with the increased medical care required for respective patients. Since the growing number of co-morbid patients is a costly burden for health care systems worldwide, there is an urgent need to further clarify if inflammation interferes with treatment and if successful treatment of depression improves maladaptive inflammatory responses.

Present concepts of the role of inflammation in depression are based on randomized controlled trials employing mostly patients suffering from major depressive disorder (15). In these studies, depression is associated at it's peak with high levels of general inflammation markers such as C-reactive protein (CRP) as well as high levels of pro-inflammatory cytokines such as tumor necrosis factor alpha (TNFα) and interleukin 6 (IL6). In a number of pharmacologic and behavioral studies, the successful treatment of depression resulted in simultaneous normalization of depression and cytokine levels (16–22). However, there is considerable variation in reports linking depression and inflammation (22) and little is known about the effects in patients with moderate depression scores or the response to other treatment concepts. Due to the observation that patients with major depression and high levels of inflammation markers appear to be more resistant to pharmacologic or behavioral treatment (11, 20, 22–26), this topic warrants further investigation of the role of inflammation in different therapeutic approaches and patient subpopulations.

Some of the variation observed between studies may be accounted for by the types of inflammatory molecules measured and the sampling material employed. For instance, many studies employed CRP measurement in serum or plasma. Increased CRP levels in depression is a rather late event and marks a persistent pro-inflammatory state. Prior to the development of a pro-inflammatory state the examination of peripheral mononuclear cell (PBMC) cytokine production is an excellent method to measure pro-inflammatory immune responses. These cells can be extracted from patient blood samples and challenged *ex vivo* by immune activators such as phytohaemagglutinin (PHA) to study, if the immune response to challenge is hyper-reactive. Among the cytokines released by PBMCs, several studies have revealed the importance of TNFα as a possible early biomarker for immune system hyper-reactivity in depressed patients. TNFα was shown in a meta-analysis to interfere with depression treatment success more frequently than other cytokines (20). TNFα was also shown to participate both in innate and adaptive immune responses that are disrupted in depressed patients with non-communicable disease. In addition, this cytokine can interact with the hypothalamus pituitary adrenal stress axis to modulate cortisol release with consequences for the immune response (27). Finally, biologics that neutralize TNFα activity were among the first anti-inflammatory medications established, and are now respected for their effectiveness, relative safety and anti-depressant effect (21, 28). TNFα may therefore be a useful lead marker for the study of depression treatment effects on immune system excitability.

Randomized controlled trials are best praxis to study treatment success (29). In mental health research, they commonly follow a single therapeutic concept and are tailored to treat a specific mental condition in a selected outpatient population. This approach is highly efficient to prove the efficacy of the therapeutic concept in question for a defined aspect of mental health. In reality, however, patient's health issues are often multi-dimensional. In routine clinical care settings this issue is met by the combination of therapeutic approaches recommended by evidence-based medicine guidelines. Within the framework of the compulsory national health insurance of the German healthcare system for example, psychotherapy is covered by insurance companies if the hospital adheres to consensus recommendations as well as a defined treatment schedule (30–34). The resulting multimodal psychotherapeutic inpatient therapy (mPT) can be based on the behavioral orientation as well as the psychodynamic orientation. It is mandatory that it comprises at least three individual and group psychotherapy sessions per week in combination with at least one session of physiotherapy, art therapy and music therapy. In addition, it is mandatory that patients receive 24 h medical care, interact with specialized nurses at least three times a day, and interact with a multi-professional supervised team of doctors and psychotherapists at least once a day. In psychosomatic medicine, mPT is often given to patients with moderate depression for which sufficient ambulatory care is not available, or depression that can be expected to improve in a psychotherapeutic setting after removal from a patients home environment. There is, however, a distinct lack of studies that address the effects of mPT and it's interaction with inflammation (14, 16, 35).

Taken together, the interaction between moderate depression and inflammation in a routine clinical care setting focussing on the psychodynamic concept has yet to be studied (14, 16, 35–37). In the present naturalistic study, PBMC cytokine production after PHA challenge was therefore analyzed in 73 patients with the primary diagnosis ICD10 F32.1/2 or F33.1/2 that received mPT at a university hospital with psychodynamic orientation. Prediction of mPT outcome by baseline PBMC cytokine production was studied in order to learn if a potential hyper-reactivity of PBMC to PHA challenge can interfere with the treatment. In addition, patients were categorized at baseline into high TNFα-producers (hTNF) or low TNFα-producers (lTNF) to illustrate treatment effects, and three analyses were performed using this design: baseline differences between hTNF and lTNF groups, changes over time in response to therapy within each TNFα group, and differences in mPT outcome between hTNF and lTNF patients.

## Materials and Methods

### Ethical Considerations and Recruitment of Participants

A naturalistic study was conducted in accordance with the Code of Ethics of the World Medical Association (Declaration of Helsinki) and approved by the ethics committee of the Justus-Liebig University, Gießen, Germany. Recruitment and assessment of study volunteers followed standardized procedures. Briefly, all patients successively admitted to the department of psychosomatic medicine at the Justus-Liebig-University in Gießen, Germany for mPT between December 2011 and February 2014 were asked permission to take a blood sample and to self-report stress and mental health. All enrolled patients provided written informed consent. We analyzed patients with depression according to ICD-10 diagnostic criteria, defined by F32.1/2 depressive episode and F33.1/2 recurrent depressive disorder. None of the patients had additional diagnosis of anxiety disorders or post traumatic stress disorder. Patients in inpatient treatment were allowed to continue with their pre-admission medication and tea, coffee or nicotine consumption. Patients with alcohol and drug issues were only assigned to psychosomatic inpatient treatment if they had stopped consumption for a considerable time prior to hospital admission. Current substance dependency was a criterion for exclusion. Of the 250 patients treated during that time, patients with other diagnosis (*N* = 112), mPT <3 weeks or incomplete questionnaires (*N* = 49), and patients with CRP higher than 14.93 mg/dl (indication of acute infection, mean+3× sd all depressives T1, *N* = 6) were excluded from the analysis. A remaining total of 73 patients were included in the analysis.

### Multimodal Psychotherapeutic Inpatient Therapy (mPT) Following a Psychodynamic Concept

The Department of Psychosomatics and Psychotherapy at the Justus Liebig University in Giessen is a German university hospital that has an inpatient unit, a day care center, and an outpatient clinic (including special outpatient clinics and a consultation and liaison service) treating approximately 400, 300, and 1,400 patients per year, respectively. The clinic follows a psychodynamic concept, complemented by targeted elements of behavioral therapy following a fixed weekly schedule. The therapeutic approach connects psychodynamic psychotherapy-based individual and group therapy, psychoeducation, music therapy, art therapy, movement therapy including elements of qigong, learning theory-based behavioral training for active stress management, autogenic training, progressive muscle relaxation according to Jacobson and functional relaxation, social work, and medical treatment according to consensus guideline recommendations. The clinical and scientific activities of the clinic focus on the therapy of people with various psychosomatic disorders, functional physical complaints, pain, primarily physical illnesses with accompanying mental health problems (e.g., cancer, diabetes, skin diseases), life crises, depression, anxiety disorders, eating disorders and post-traumatic stress disorder. Patients' admission for psychotherapeutic inpatient treatment does not depend on the severity of their disease but is initiated when it is believed that sufficient disease control can only be achieved in the inpatient setting (32). Besides the symptoms, the conditions that lead to symptom development, co-morbidities, treatment goals, motivation for treatment, structure of patient personality, professional, social and family situation, and the patient's treatment wishes are taken into account. In German psychosomatic medicine, these criteria often apply to patients with moderate depression and high psycho-social stress while patients with severe depression and suicidal ideation are mostly treated in psychiatric clinics.

### Blood Sample Acquisition and Processing

Patients were asked to refrain from coffee, tea, nicotine or physical activity on the morning of blood collection. Blood samples were obtained between 8 and 9 o'clock in the morning on the first and last day of the hospital stay and immediately processed. Serum was obtained and stored at −80°C for future analysis. PBMC were harvested by Ficoll separation, which allows for the isolation of all nucleated cells in the blood sample. 1.25 × 10^6^ PBMC per patient and timepoint were stimulated with phytohaemagglutinin (PHA, Sigma-Aldrich, St. Louis, MO, USA) in Aim V medium at 37°C/5% CO_2_. After 24 h of stimulation, supernatants were collected and stored at −80°C for future analysis. PHA was chosen because it is a lectin from phaeseolus vulgaris, which is widely used in immunological studies to promote proliferation and provoke cytokine release from PBMC for example in depressive patients (38, 39). PHA was also chosen, because it acts on a wide variety of cells of the immune system. Since distribution of PBMC subpopulations can differ greatly between individuals but all cell populations respond to a non-specific challenge, all PBMC can be expected to contribute to a potential hyper-inflammatory response. For this reason, measurement of overall cytokine production by challenged PBMC illustrates the overall reactivity of a patient's immune cells in the circulation to such a challenge.

### Determination of Cytokines With Luminex xMap Technology *in vitro*

Cytokines in cell culture supernatant of all patients in the study were measured using a bio-plex pro human cytokine 11-plex assay (IL1β, IL2, IL4, IL5, IL6, IL10, IL12p70, IL17A, eotaxin, interferon gamma [IFNγ] and TNFα (Biorad Laboratories, Munich, Germany) optimized according to routine standard procedures by testing duplicates. To ensure optimal sample comparison, each 96 well plate for cytokine analysis contained samples from both patient groups and care was taken to include all time points from an individual on the same 96 well plate. The lower limit of detection (in pg/ml) and inter-assay coefficient of variation were as follows: IL1β: 0.6/8, IL2: 1.6/9, IL4: 0.7/8, IL5: 0.6/10, IL6: 2.6/11, IL10: 0.7/6, IL12p70: 3.5/6, IL17: 3.3/6, Eotaxin: 2.5/11, IFNγ: 6.4/9, TNFα: 6/6. The reliability of Biorad multiplex cytokine assays has been validated in the following publication (40). A Bio-Plex 200 System was used as recommended by the manufacturer (Bio-Rad) and concentrations were calculated using Bio-Plex Manager Software 6.

### ELISA Determination of Broad Inflammation Markers

Enzyme-linked immunosorbent assay (ELISA) was used to detect serum levels of CRP, HMBG1, leptin. HMGB1 and leptin (IBL International GMBH, Hamburg, Germany) as recommended by the respective manufacturers. The intra-assay coefficients of variation for all reported ELISAs were <3% and all results measured were within the detection range.

### Self-Report Assessment

The subscales “depression” and “anxiety” of the Hospital Anxiety and Depression Scale (HADS) were used to assess symptom load and delta HADS “depression” subtracting T2 from T1 levels to assess treatment success. Test quality criteria of the employed questionnaire are described in the following citation (41).

### Power Analysis

Power analysis for the main hypothesis was performed with G^*^Power 3.1. We calculated the numbers of subjects that are needed to calculate a regression with a power of 0.80 at an a-level of 0.05 assuming a small to medium effect size of 0.1 as commonly found in quasi-experimental naturalistic studies in psychotherapy research. This resulted in a required power of *N* = 73.

### Statistical Analysis

Statistics for Windows software (SPSS), version 24 (Armonk, NY, United States) was used for the statistical evaluation. The nominal item sex was converted into the dummy variables male = 1, female = 2. Missing cytokine values were replaced with the half of the lowest values when cytokines were detectable but below the quantification limit as recommended by the manufacturer (<10% of all measurements). For psychometric assessments, ordinal items were converted to a scale ranging from 0 to 100 points where applicable. The category “does not apply” and item non-response were coded as missing values. Logarithmic transformations to achieve approximately normal data distribution were performed where applicable.

To investigate the relationship between TNFα baseline production and mPT outcome defined as delta HADS “depression” (T1–T2), multiple linear regression models were used, controlling for potential confounders (e.g., age and sex) (42). This allowed us to reject the null hypothesis that baseline TNFα levels are irrelevant for treatment outcome (see results section Ethical considerations and recruitment of participants).

In addition to the regression analysis, the Median-Split-Method was used to separate the participants into two categories according to PBMC TNFα production at baseline (T1) with the aim to illustrate characteristics of patients with high TNFα production and to learn what measurements besides the main outcome HADS ‘depression’ showed improvement after treatment in high TNFα producers. Ranking was hence done by a separate variable than the main outcome and based on data assessed prior to treatment rather than dividing the group in treatment responders and non-responders *post-hoc*. Participants with TNFα values ≥29.04 pg/ml were grouped as high TNFα producers (hTNF) and all others as low TNFα producers (lTNF). All metric values were calculated as mean and standard deviation (sd) per group and time point. According to group assignment, baseline analysis of differences in socio-demographic and clinical data between the lTNF and hTNF groups was done by Chi-X2-Test for ordinal data and by Mann–Whitney *U*-test for metric data. For hTNF and lTNF, group comparisons of laboratory and self-report data at baseline and after mPT, Students *t*-tests for independent samples were used. To compare mean values of laboratory and self-report data at baseline and after therapy within the groups, paired *t*-tests with repeated measures were used. As TNFα was not the main outcome and a Median and not a Mean Split was performed, regression to the mean was not considered an issue with respect to the main outcome HADS “depression” and all other measures besides TNFα. Because of the small number of participating patients, a bootstrapping technique was used to test for the significance of all *t*-tests. This treats a given sample as the population using intensive computer resampling (1,000 sampling) (43, 44). *P* < 0.05 were considered significant. Effect sizes were computed using Cohen's d.

To analyse potential interactions between developments over time in the two subgroups, two way ANOVAs were calculated.

## Results

### Is Baseline TNFα Associated With mPT Outcome?

We found that baseline TNFα production of PBMC in response to PHA challenge predicted mPT outcomes, and higher logTNFα at T1 (baseline) predicted higher improvement of HADS “depression” (delta HADS “depression” = T2 (discharge) scores—T1 (admission) scores) in the total patient sample. This analysis proved to be robust with respect to various confounders included in three different models (for details on models see legend to Table 1). However, the *R*^2^ was relatively low in all models (Table 1). High IFNγ and IL10 also predicted better HADS “depression” outcome, albeit with lower *R*^2^ than TNFα in the confounder controlled model (not shown). Other cytokines and general inflammation markers (CRP, HMGB1, leptin) did not predict outcome (not shown) whereby the levels of general inflammation markers were generally low.

**Table 1:** Prediction of ΔHADS “depression” by Log TNFα. Uni- and multivariate regression analysis in confounder controlled models.

	Model I^*^	Model II^**^	Model III^***^
	**(1, 68)**	**(4, 68)**	**(6, 64)**
F	5.315	2.501	1.866
R^2^	0.075	0.135	0.162
B	−0.828	−0.865	−0.928
Beta	−0.271	−0.283	−0.321
*p*	**0.024**	**0.022**	**0.014**

* Unadjusted;

** adjusted for BMI, sex, age;

*** adjusted for BMI, sex, age, diagnosis, treatment with anti-depressants.

*All values, which have a p < 0.05 are printed bold to highlight significant differences*.

### Do Depressive Patients With High TNFα Production at Baseline Differ From Patients With Low Baseline TNFα Production With Respect to General Population Characteristics?

Further analysis was conducted after splitting the sample into hTNF and lTNF patients. The question was, if hTNF showed e.g., higher BMI or lower socio-educational status as reported in other studies reporting a pro-inflammatory status in depression. Concerning baseline data at admission, no differences between the groups were detected with respect to diagnosis subtype distribution (ICD10 F32.1/2, F33.1/2), weeks of mPT, age, sex, BMI, family situation, or education. Also, no difference was found with respect to medication, which was generally low in both groups (e.g., only 10/37 and 15/36 took anti-depressants) (Table 2). Hence, clinically hTNF and lTNF groups were not discernable prior to therapy.

**Table 2:** Baseline comparison of socio-demographic and clinical data^*^.

**Parameter**	**Details**	**hTNF**	**lTNF**	**(Comparison of means**
	**(N hTNF/N lTNF)**	**(high PBMC TNF production group)**	**(low PBMC TNF production group)**	**as indicated in legend)**
		**Mean (sd)**	**Mean (sd)**	***p***
Age		43.47 (12.67)	38.38 (12.46)	0.086
Sex	male (36/37)	13 (36.1%)	11 (29.7%)	0.80
	female (36/37)	23 (63.9%)	26 (70.3%)	
BMI		26.56 (5.74)	26.69 (7.74)	0.90
Family	in partnership (35/35)	23 (65.7%)	22 (62.9%)	0.80
	married (35/36)	11 (31.4%)	11 (30.6%)	0.79
	with children (35/36)	22 (62.9%)	20 (55.6%)	0.47
Education	secondary modern school (35/36)	10 (28.6%)	9 (25.0%)	0.59
	junior high school (35/36)	14 (40.00%)	16 (44.4%)	0.63
	university-entrance diploma (35/36)	11 (31.4%)	11 (30.6%)	> 0.99
Diagnosis	F32.1 (36/37)	35 (97.2%)	36 (97.3%)	> 0.99
	F33.2 (36/37)	1 (2.8%)	1 (2.7%)	
Weeks of mPT		7.37 (2.34)	6.73 (2.30)	0.35
Medication	tranquilizer (36/37)	2 (5.6%)	0 (0.0%)	0.15
	anti-depressants (36/37)	15 (41.7%)	10 (27.0%)	0.077
	neuroleptics (36/37)	0 (0.0%)	1 (2.7%)	0.32
	opiods (36/37)	0 (0.0%)	0 (0.0%)	–
	NSAR (36/37)	4 (11.1%)	3 (8.1%)	0.69
	anti-epileptics (36/37)	2 (5.6%)	3 (8.1%)	0.64
	other (36/37)	20 (55.6%)	15 (40.5%)	0.23

* *Metric data were compared by Mann–Whitney U-test, ordinal data by Chi-X2-Test*.

### Do Depressive Patients With High TNFα Production at Baseline Differ From Patients With Low Baseline TNFα Production With Respect to Inflammation and Mental Health Markers?

The first question here was, if hTNF patients showed a general hyper-responsiveness to PHA challenge, or if certain aspects of the immune response such as acute inflammatory markers were selectively upregulated, as suggested by a number of previous studies on pro-inflammatory cytokines in depression. Depressive patients in the hTNF group showed significantly higher PBMC cytokine production than the lTNF group. Significantly increased cytokine levels in hTNF patients with medium to high effect sizes included IL1β and IL6 (acute inflammatory cytokines), IFNγ [T-helper cell type (TH) 1 cytokine], IL17A (TH17 cytokine), IL4 (TH2 cytokine), eotaxin (eosinophilic inflammation), and IL2 (regulation of inflammation) (Table 3A). However, with respect to general markers of pro-inflammatory status (serum CRP, HMGB1, and leptin), no significant difference was seen between hTNF and lTNF groups and CRP levels were generally low and in the non-pathologic range though a tendency toward a slightly higher CRP was present in hTNF (Table 3B).

**Table 3:** Baseline comparison of hTNF and lTNF depressive patients^*^.

		**hTNF** ** (high PBMC TNF** **production group)** ** (*N* = 36)**	**lTNF** ** (low PBMC TNF** ** production group)** ** (*N* = 37)**		
		**Mean (sd)**	**Mean (sd)**	**p**	**d**
**A: Cytokines produced by PHA stimulated PBMC**					
Acute inflammatory response cytokines					
	TNFα (pg/ml)	282.47 (488.33)	13.94 (7.54)	**0.001**	**−0.783**
	IL1β (pg/ml)	44.99 (114.05)	2.40 (3.08)	**0.026**	**−0.532**
	IL6 (pg/ml)	1,371.99 (3,171.64)	51.69 (120.81)	**0.014**	**−0.592**
NK and TH1 cell promoting cytokines					
	IL12p70 (pg/ml)	3.92 (5.36)	2.21 (2.11)	0.076	−0.422
TH1 cytokines					
	IFNγ (pg/ml)	55.82 (78.37)	3.65 (6.20)	** <0.001**	**−0.945**
TH17 cytokines					
	IL17A (pg/ml)	9.64 (13.60)	1.47 (3.42)	**0.001**	**−0.828**
TH2 cytokines					
	IL4 (pg/ml)	0.90 (1.37)	0.05 (0.11)	** <0.001**	**−0.879**
	IL5 (pg/ml)	1.02 (1.62)	0.64 (0.63)	0.18	−0.313
Eosinophil chemotactic cytokines					
	Eotaxin (pg/ml)	7.02 (8.13)	1.14 (1.54)	** <0.001**	**−1.013**
Inflammation regulating cytokines					
	IL2 (pg/ml)	14.63 (22.39)	2.14 (2.73)	**0.001**	**−0.789**
	IL10 (pg/ml)	9.16 (24.83)	1.36 (0.90)	0.060	−0.447
**B: Broad biomarkers of systemic inflammation in serum**					
	CRP (mg/dl)	2.16 (3.00)	1.12 (1.67)	0.078	−0.429
	HMGB1 (ng/ml)	3.54 (3.17)	3.53 (2.63)	0.87	−0.005
	Leptin (ng/ml)	10.93 (8.40)	13.15 (14.80)	0.50	0.184
**C: Self-report data**					
HADS	“Summary score”	22.38 (7.85)	22.20 (6.65)	0.73	−0.025
	“Anxiety”	11.05 (4.60)	10.70 (4.24)	0.86	−0.079
	“Depression”	11.32 (4.56)	11.50 (4.21)	0.86	0.041

* *Metric data were compared by students t-test for independent samples with bootstrap method (1,000 sampling). Please note that the cut-off for moderate depression is 11, the cut-off for major depressive disorder is 14/15 (45; 46)*.

*All values, which have a p < 0.05 are printed bold to highlight significant differences*.

Second, we wanted to know, if hTNF patients were suffering from more depressive symptoms. Using a representative self-report instrument to assess mental health in the dimensions anxiety and depression (HADS), no significant differences were found between hTNF and lTNF patients with respect to disease severity at baseline and the mean in both groups corresponded to moderate depression (45, 46) (Table 3C).

### Do Markers of Inflammation and Mental Health Change Over Time in Response to mPT?

**Figure 1:**
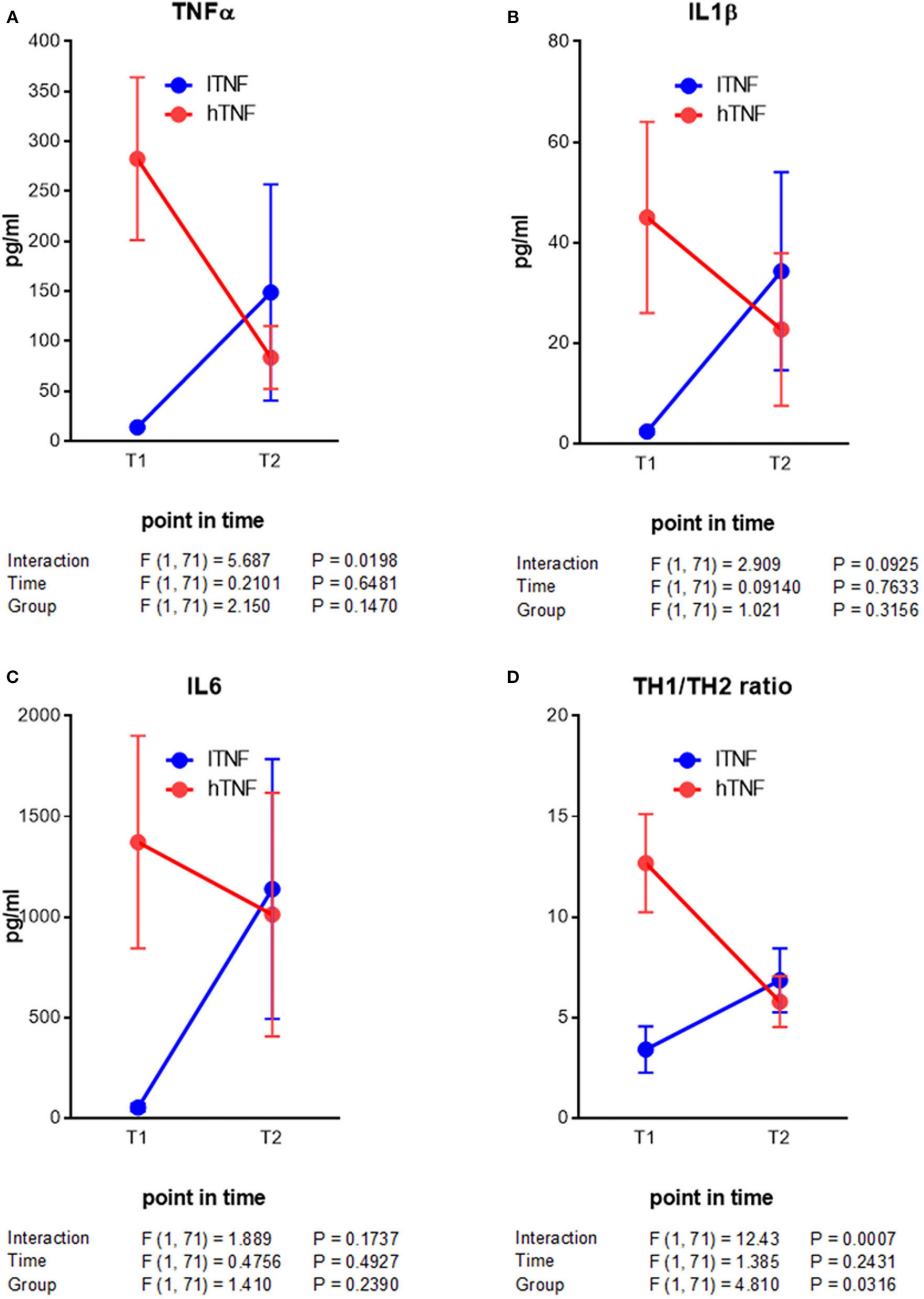
Analysis of interaction of contrarious cytokine level developments in depressive patients after mPT. **(A–C)** Acute inflammatory response cytokines. **(D)** TH1/TH2 ratio. Results of statistical analysis are given below the graphs.

Here we were interested to learn about improved outcomes with respect to PBMC cytokine production and mental health. From the beginning to the end of therapy, hTNF patients showed a decrease in all measured PBMC cytokines, with significant reductions in IFNγ, IL4 and eotaxin (Table 4A). In lTNF patients we observed significant differences in the TH1/TH2 ratio. With respect to broad markers of inflammation (CRP, HMGB1, and leptin), no significant changes were seen in the total group or in either hTNF or lTNF patients from the beginning to the end of therapy (Table 4B). Finally, symptoms of anxiety and depression (HADS) improved highly significantly in both hTNF and lTNF groups from the beginning to the end of therapy, whereby larger effect sizes were observed in the hTNF group (*d* = 1.1–1.3) than the lTNF group (*d* = 0.6–0.9) (Table 4C). Additionally we performed a two way ANOVA analysis of key immune measures. This revealed a significant time by group interaction for TNFα and TH1/TH2 ratios (Figure 1).

**Table 4:** Timewise comparison of baseline vs. mPT outcome in hTNF and lTNF groups^*^.

**A: Cytokines produced by PHA stimulated PBMC**		**Timepoint 1** ** (admission)**	**Timepoint 2** ** (discharge)**		
**hTNF (high PBMC TNF production group)**		**Mean (sd)**	**Mean (sd)**	***p***	***d***
**Acute inflammatory response cytokines**					
	TNFα (pg/ml)	282.47 (488.33)	83.59 (188.68)	0.056	−0.285
	IL1α (pg/ml)	44.99 (114.05)	22.70 (91.07)	0.40	−0.134
	IL6 (pg/ml)	1,371.99 (3,171.64)	1,011.31 (3,636.72)	0.69	−0.077
**NK and TH1 cell promoting cytokines**					
	IL12p70 (pg/ml)	3.92 (5.36)	2.61 (2.01)	0.18	−0.185
**TH1 cytokines**					
	IFNγ (pg/ml)	55.82 (78.37)	20.53 (53.73)	**0.029**	**−0.328**
**TH17 cytokines**					
	IL17A (pg/ml)	9.64 (13.60)	4.59 (11.07)	0.077	−0.278
**TH2 cytokines**					
	IL4 (pg/ml)	0.90 (1.37)	0.34 (0.90)	**0.038**	**−0.306**
	IL5 (pg/ml)	1.02 (1.62)	0.93 (2.01)	0.79	−0.045
**Eosinophil chemotactic cytokines**					
	Eotaxin (pg/ml)	7.02 (8.13)	3.34 (5.74)	**0.032**	**−0.320**
**Inflammation regulating cytokines**					
	IL2 (pg/ml)	14.63 (22.39)	4.11 (5.26)	0.070	−0.389
	IL10 (pg/ml)	9.16 (24.83)	3.65 (8.40)	0.33	−0.160
**TH1/TH2-ratio**					
	IFNγ/IL10^*^	12.66 (14.66)	5.77 (7.56)	**0.023**	**−0.345**
**lTNF (low PBMC TNF production group)**					
**Acute inflammatory response cytokines**					
	TNFα (pg/ml)	13.94 (7.54)	148.69 (657.27)	0.38	11.733
	IL1β (pg/ml)	2.40 (3.08)	34.29 (119.83)	0.24	7.145
	IL6 (pg/ml)	51.69 (120.81)	1,139.04 (3,933.74)	0.22	6.333
**NK and TH1 cell promoting cytokines**					
	IL12p70 (pg/ml)	2.21 (2.11)	2.89 (2.88)	0.15	0.305
**TH1 cytokines**					
	IFNγ (pg/ml)	3.65 (6.20)	26.99 (72.98)	0.17	2.585
**TH17 cytokines**					
	IL17A (pg/ml)	1.47 (3.42)	5.41 (12.91)	0.13	0.845
**TH2 cytokines**					
	IL4 (pg/ml)	0.05 (0.11)	0.46 (1.22)	0.14	2.572
	IL5 (pg/ml)	0.64 (0.63)	0.91 (1.01)	0.052	0.520
**Eosinophil chemotactic cytokines**					
	Eotaxin (pg/ml)	1.14 (1.54)	4.34 (7.76)	0.072	1.358
**Inflammation regulating cytokines**					
	IL2 (pg/ml)	2.14 (2.73)	3.48 (5.48)	0.11	0.491
	IL10 (pg/ml)	1.36 (0.90)	3.93 (10.97)	0.37	2.040
**TH1/TH2-ratio**					
	IFNγ/IL10^*^	3.40 (7.04)	6.84 (9.63)	**0.016**	**0.553**
**B: Broad biomarkers of systemic inflammation in serum**		**Timepoint 1** ** (admission)**	**Timepoint 2** ** (discharge)**		
**hTNF (high PBMC** ** TNF production group)**		**mean (sd)**	**mean (sd)**	***p***	***d***
	CRP (mg/dl)	2.16 (3.00)	2.13 (3.97)	0.96	−0.012
	HMGB1	3.55 (3.18)	3.49 (2.61)	0.87	−0.025
	Leptin	10.93 (8.40)	11.34 (8.61)	0.59	0.094
**lTNF (low PBMC TNF** ** production group)**					
	CRP (mg/dl)	1.15 (1.67)	1.27 (1.80)	0.46	0.133
	HMGB1	3.43 (2.66)	2.99 (2.35)	0.17	−0.209
	Leptin	12.84 (14.72)	13.59 (13.08)	0.37	0.150
**C: Self-report data**		**Timepoint 1** ** (admission)**	**Timepoint 2** ** (discharge)**		
**hTNF (high PBMC TNF** ** production group)**		**mean (sd)**	**mean (sd)**	***p***	***d***
HADS	“Summary score”	22.38 (7.85)	12.15 (8.80)	**0.001**	**−1.299**
	“Anxiety”	11.05 (4.60)	6.68 (4.82)	**0.001**	**−1.135**
	“Depression”	11.31 (4.56)	5.47 (4.67)	**0.001**	**−1.184**
**lTNF (low PBMC TNF** ** production group)**					
HADS	“Summary score”	22.20 (6.65)	15.38 (7.97)	**0.001**	**−0.899**
	“Anxiety”	10.70 (4.24)	7.46 (3.74)	**0.002**	**−0.693**
	“Depression”	11.50 (4.21)	7.92 (5.04)	**0.001**	**−0.765**

* *Admission levels were compared to demission levels. Metric data were compared by paired t-test for independent samples with bootstrap method (1,000 sampling)*.

*All values, which have a p < 0.05 are printed bold to highlight significant differences*.

### Does Depression at Discharge Differ Between TNF Groups?

Finally, we wanted to know the extent to which inflammation markers and mental health improved in hTNF and lTNF. After therapy, differences between hTNF and lTNF groups could no longer be detected with respect to cytokines (Table 5A), while broad markers of inflammation remained without difference (Table 5B). By contrast, the HADS “depression” score was significantly lower in hTNF patients compared to lTNF patients, which remained close to moderate depression cut-off (Table 5C).

**Table 5:** Comparison of hTNF and lTNF depressive patients after mPT^*^.

		**hTNF** ** (high PBMC TNF** ** production group)** ** (*N* = 36)**	**lTNF** ** (low PBMC TNF** ** production group)** ** (*N* = 37)**		
**A: Cytokines produced by PHA stimulated PBMC**		**mean (sd)**	**mean (sd)**	***p***	***d***
Acute inflammatory response cytokines					
	TNFα (pg/ml)	83.59 (188.68)	148.69 (657.27)	0.56	0.134
	IL1β (pg/ml)	22.70 (91.07)	34.29 (119.83)	0.64	0.109
	IL6 (pg/ml)	1,011.31 (3,636.72)	1,139.04 (3,933.74)	0.88	0.034
NK and TH1 cell promoting cytokines					
	IL12p70 (pg/ml)	2.61 (2.01)	2.89 (2.88)	0.63	0.112
TH1 cytokines					
	IFNγ (pg/ml)	20.52 (53.72)	26.99 (72.98)	0.66	0.101
TH17 cytokines					
	IL17A (pg/ml)	4.59 (11.06)	5.40 (12.90)	0.77	0.067
TH2 cytokines					
	IL4 (pg/ml)	0.34 (0.90)	0.46 (1.22)	0.62	0.112
	IL5 (pg/ml)	0.92 (2.00)	0.90 (1.01)	0.96	−0.013
Eosinophil chemotactic cytokines					
	Eotaxin (pg/ml)	3.33 (5.74)	4.33 (7.76)	0.53	0.146
Inflammation regulating cytokines					
	IL2 (pg/ml)	4.11 (5.25)	3.47 (5.48)	0.61	−0.119
	IL10 (pg/ml)	3.65 (8.39)	3.92 (10.97)	0.90	0.028
**B: Broad biomarkers of systemic inflammation in serum**					
	CRP (mg/dl)	2.13 (3.97)	1.27 (1.80)	0.23	−0.280
	HMGB1	3.49 (2.61)	2.98 (2.35)	0.38	−0.206
	Leptin	11.34 (8.61)	13.59 (13.08)	0.38	0.203
**C: Self-report data**					
HADS	“Summary score”	12.56 (8.83)	15.38 (8.00)	0.10	0.335
	“Anxiety”	6.68 (4.83)	7.46 (3.74)	0.44	0.181
	“Depression”	5.47 (4.67)	7.92 (5.04)	**0.035**	**0.504**

A: Cytokine production by PHA stimulated PBMC. B: Systemic inflammatory mediators measured in serum. C: Symptoms of and anxiety measured by HADS. Of note: data reported here are comparable to TNF levels produced by PBMC previously reported in healthy participants (47–49).

* *Metric data were compared by students t-test for independent samples with bootstrap method (1,000 sampling)*.

*All values, which have a p < 0.05 are printed bold to highlight significant differences*.

## Discussion

We report a significant response to multimodal psychotherapeutic inpatient therapy (mPT) following a psychodynamic concept in patients with moderate depression and high production of TNFα by PBMC at the time of hospital admission. In addition, when the patients were assigned to a high TNFα producing subgroup (hTNF) and compared to a low TNFα producing subgroup (lTNF), the hTNF exhibited a distinct PBMC cytokine production profile in response to PHA challenge, but did not differ significantly in clinical data, general inflammation markers or mental health at baseline. mPT improved mood and mental health in both subgroups. Importantly however, hTNF displayed significantly higher mood improvement after therapy in comparison to lTNF. To our knowledge, this is the first time that improved cytokine production and mental health in moderate depression is reported together in a routine clinical care setting with psychodynamic orientation.

This outcome contrasts a number of studies reporting treatment resistance in major depressive patients with high TNFα levels, which requires careful discussion (3, 11, 22, 47, 50–60). In our study, we measured cytokine production by peripheral blood mononuclear cells (PBMC) stimulated *ex vivo* by a PHA challenge in a sample of patients with relatively normal general inflammation markers. Our results suggest that in the absence of pathologically increased general inflammation markers, hyper-reactivity of PBMC to an inflammatory challenge identified patients on the verge of developing a pro-inflammatory state at a point in time, where that process was well-reversible. In most previous studies addressing inflammation markers in depression, plasma or serum samples were used for assessment. In these bodily fluids, the presence of a cocktail of high concentrations of cytokines indicates *in vivo* activation of an inflammatory cascade. If cytokine levels in serum or plasma are increased, this usually correlates with raised levels of general markers of inflammation and tissue damage such as CRP. Cytokines are thus measurable in plasma or serum, when stress-associated biomolecular processes have taken place that caused cytokines to spill-over from damaged tissue into the circulation. This consideration also provides an explanation why there is considerable variation in serum or plasma cytokine levels reported in depressive patients as patients in different phases of stress-induced inflammatory damage may have been included (22).

In this study, TNFα production by PBMCs was successfully employed as an indicator cytokine for the prediction of treatment outcome and for the description of patient subpopulations. Analysis of cytokine levels produced by PBMC may therefore be a robust method to determine a patient's inflammatory hyper-reactivity to challenge prior to damage development. It is promising that hTNF patients are especially responsive to mPT and encourages longitudinal studies investigating the risk of long-term co-morbid disease development after treatment in these patients. The results also argue for the necessity of intervention in patients with moderate depression and that these patients may profit from additional anti-inflammatory treatment. At the same time, it can be hypothesized that patients that show little responsiveness of their PBMC to challenge require intensified treatment.

In support of the concept that subpopulations of depression patients can be defined by inflammatory activity, many studies report higher baseline cytokine levels in depressives prior to treatment when compared with healthy controls. Furthermore, these studies reveal that after treatment, depressives and healthy controls no longer differ in their cytokine levels (3, 22, 53, 60–65). Interestingly, the reported levels of cytokines in these studies prior to treatment were similar to the levels observed in our hTNF patients prior to mPT. Also, studies in which baseline cytokine levels in depressives did not differ from healthy controls, reported an increase in pro-inflammatory cytokines after treatment similar to our observation in lTNF (66, 67). It is therefore feasible to argue that there are at least two subpopulations of patients with depression with regard to their immune responsiveness, one that is hyper-reactive to challenge and well-treatable and another that is immunologically innate, not challengeable and more resistant to therapy.

Due to the naturalistic hospital setting, the lack of healthy controls or untreated control patients, the unavailability of a randomized controlled design, and successive recruitment are clear limitations of our study. Also, the possibility of a regression to the mean has to be considered, when looking at dichotomized data. In an experimental set up providing data that may depend on many variables, chance can be involved and it is to be ruled out that extreme outcomes observed at one point in time are followed by more moderate ones at another by chance and not due to a real improvement. If regression to the mean were the case, the measures observed in our patients prior to mPT would not differ from measures previously reported in healthy populations and the standard deviations in all analyzed subpopulations would be larger at T2 compared to T1 (68). However, our here analyzed data set did not fulfill these criteria for regression to the mean and allowed the comparison of hTNF and lTNF patients under real life conditions in the light of published data on healthy cytokine production by PBMC.

Also to be considered, one study reported that patients responsive to treatment displayed higher baseline cytokine levels than patients non-responsive to treatment (69). Hence, alternative to the approach employed in our study, response to treatment could have been used to subdivide study populations and analyze characteristics of patients with unfavorable treatment outcome. However, treatment response can only be determined *post-hoc*, while assessment of responsiveness to immune challenge can distinguish subgroups prior to treatment. Of course, the technical requirements for PBMC isolation and *ex vivo* challenge with PHA are rather high and may require a university hospital setting with a fully equipped immunology laboratory at hand. Though compared to flow cytometry for the identification of PBMC subpopulations, the determination of cytokines in cell culture supernatants can be done with comparably smaller effort. Future studies in this vein could establish this more complex but highly instructive analysis side by side with more easily applicable protocols to determine hyper-reactivity of the immune system to challenge in the clinic, for example by using skin tests (70).

Another consideration is that it has been known for some time that exposure to traumatizing experiences as well as substantial challenges to the immune system during early childhood may contribute not only to posttraumatic stress disorder and depression but also to lifelong changes in neuroendocrine and inflammatory responsiveness (71, 72). Recent research has found that, being traumatized reduced the adaptive capacity of the neuroendocrine and immune systems to interact efficiently in response to a new challenge (73–75). However, the changes resulting from traumatization tend to be lasting and can be expected to increase systemic inflammation markers (76). As we did not observe this, and our patient population showed impact of event scale scores below the cut-off of 26 (data not shown), this consideration may not play a role in our patient sample.

In summary, analysis of PBMC cytokine production provides a robust view of immune system reactivity that links high TNFα production in moderately depressive patients at baseline with improved mPT treatment outcome. We report normalization of cytokine production in response to challenge in hTNF after mPT, demonstrating that depression-associated changes in immune system function are reversible in this subpopulation of patients in a clinical psychosomatic inpatient care setting employing multimodal psychotherapeutic inpatient therapy focusing on a psychodynamic concept. This suggests that mPT is effective both on the mental and the somatic level, which contributes to the understanding of psychoimmune circuits involved in depression. Our findings in patients subjected to mPT suggest that the capacity of cells of the immune system to produce inflammatory cytokines can indicate greater adaptive responsiveness of the affected depressive patients to therapy and suggest a more complex relationship between inflammation and depression than previously hypothesized.

## Data Availability Statement

The raw data supporting the conclusions of this article will be made available by the authors, without undue reservation.

## Ethics Statement

The studies involving human participants were reviewed and approved by Ethics committee of the Justus-Liebig University, Gießen, Germany. The patients/participants provided their written informed consent to participate in this study.

## Author Contributions

EP conceptualized and designed the study. MN and AM recruited patients and collected the biological samples under the supervision of EP, SA, FLei, and MC. MN generated the cytokine data with support from AM and under the supervision of MC. MN, AM, and ST collected all other data. EP, MN, and ST performed data processing and statistical analysis. MR and JK provided general support. EP, MC, and JK provided funding and supervised all stages of the study. MR, SA, FLew, and JK provided clinical supervision for the study. EP, MN, and ST interpreted the data. EP drafted the manuscript and coordinated the manuscript writing. All authors read, revised critically, and approved the final manuscript.

## Conflict of Interest

The authors declare that the research was conducted in the absence of any commercial or financial relationships that could be construed as a potential conflict of interest.
